# The potential protective effect of Camellia *Sinensis* in mitigating monosodium glutamate-induced neurotoxicity: biochemical and histological study in male albino rats

**DOI:** 10.1007/s11011-024-01365-0

**Published:** 2024-06-13

**Authors:** Walaa G. Abdelhamid, Noha A. Mowaad, Gihan F. Asaad, Asmaa F. Galal, Sarah S. Mohammed, Olfat E. Mostafa, Doaa R. Sadek, Lobna A. Elkhateb

**Affiliations:** 1 Forensic Medicine and Clinical Toxicology Department, Faculty of Medicine, Ain Shams University, Cairo, Egypt; 2 Narcotics, Ergogenics and Poisons Department, Medical Research and Clinical Studies Institute, National Research Centre, Cairo, Egypt; 3 Pharmacology Department, Medical Research and Clinical Studies Institute, National Research Centre, Cairo, Egypt; 4 Biochemistry Department, Poison Control Center, Ain Shams University Hospitals, Cairo, Egypt; 5 Histology Department, Faculty of Medicine, Ain Shams University, Cairo, Egypt

**Keywords:** Antioxidants, Calretinin, GFAP, Monosodium glutamate, Neurotoxicity, Neurotransmitters

## Abstract

Monosodium glutamate (MSG) is the sodium compound derived from glutamic acid. Excessive daily ingestion of MSG leads to elevated amounts of glutamic acid in the bloodstream, which can be detrimental to brain structures. Camellia sinensis, often known as green tea (GT), is a rich source of essential hexogen antioxidants that are necessary for the body. Thirty-two adult male albino rats were divided into four groups (*n* = 8). Group 1 served as a control -ve group. Group 2 was given GT (1.5 ml/rat/day). Group 3 was given MSG (600 mg/kg/day). Group 4 was given MSG (600 mg/kg/day) and GT (1.5 ml/rat/day). All treatments were given orally for 28 days. MSG administration resulted in significant neurotoxicity in rats that was revealed by the significant reduction of serum concentration of glutathione peroxidase (GPx) and nitric oxide (NO), and the significant elevation of total antioxidant capacity (TAC) accompanied by the significant reduction of levels of serum monoamines (dopamine, serotonin, and norepinephrine) and histological changes in the hippocampus area CA1, dentate gyrus, and cerebellar cortex and positive immunohistochemical staining of glial fibrillary acidic proteins (GFAP) and calretinin. Administration of GT with MSG counteracted the MSG-mediated oxidative stress by significantly increasing serum concentrations of GP_X_ and NO and significantly decreasing concentrations of TAC. Furthermore, GT significantly increased levels of serum monoamines (dopamine, serotonin, and norepinephrine). Moreover, it ameliorated the histological changes, GFAP, and calretinin immunostaining in brain tissues. It is envisaged that GT will serve as a viable protective choice for the inclusion of the neurotoxicity treatment procedure.

## Introduction

Food additives are chemical substances that are included into food products with the purpose of preserving them and improving their taste and appearance. Increasing scientific data suggests that some dietary additives may pose a risk to human health. Although certain options are detrimental to one’s health and should be avoided, there are also alternatives that are considered safe and carry a negligible level of danger. Multiple studies have indicated that the presence of harmful additives and preservatives is linked to a wide range of health issues, such as obesity, cancer, ADHD, asthma, and cardiovascular difficulties (Sambu et al. 2022). In general, dietary additives have neurotoxic effects on rats, including changes in their neurological scales or scores, oxidative stress, changed neurochemical behavior, and toxic effects on their mitochondrial neurodevelopment (Biswas et al. 2022; Boyina and Dodoala 2020; Lau et al. 2006). One example of a contentious food ingredient is monosodium glutamate.

Monosodium glutamate (MSG, C5H8NO4Na) is the sodium derivative of glutamic acid. Encrypted E621. It is a commonly utilized flavor enhancer that has been employed for approximately a century in various culinary preparations, such as chips, meat items, soups, sauces, mixed condiments, and puddings. Recent study indicates that specific glutamate receptors located in the stomach and intestine play a crucial role in determining the taste and pleasantness of MSG (Torii et al. 2013). In humans, the average daily intake of total glutamate is 10 g, while the daily intake of free glutamate is greater than 1 g in various forms (Beyreuther et al. 2007). In chemical and food processing facilities, the term “MSG” encompasses all glutamic acid that is produced commercially. Excessive daily ingestion of MSG leads to elevated and accumulated amounts of glutamic acid in the bloodstream (Garattini 2000). Numerous research demonstrated the harmful consequences of daily MSG use on numerous organs (Dief et al. 2014; Farombi and Onyema 2006; Shivasharan et al. 2013).

Glutamic acid is a non-essential amino acid that serves as an important excitatory neurotransmitter in the central nervous system (CNS). Additionally, it is used as a building block for the production of glutathione and as a source of energy for some tissues (Freeman 2006). The presence of free glutamic acid can pose a problem due to its ability to interact with numerous receptors in brain tissues. Additionally, the blood-brain barrier, which is permeable in certain areas of the brain such as the hypothalamus, might further contribute to this issue (Gruenbaum et al. 2022). Thus, free glutamic acid can enter the brain through food sources and harm or frequently kill neurons. Many research points to the possibility that MSG may be neurotoxic to rats (Dief et al. 2014; Stricker-Krongrad et al. 1992; Vorhees 2018). Experimental models show that most tissue injuries caused by MSG result from oxidative stress and inflammatory pathway activation (Reifen et al. 2004; Seiva et al. 2012), therefore, more research into the molecular mechanisms underlying monosodium glutamate is required to completely understand how MSG impacts the brain. To prevent these negative consequences, scientists are therefore searching for effective natural remedies, although further study is needed. Monoamine neurotransmitters include dopamine, serotonin, epinephrine, and norepinephrine. They are also referred to as catecholamines since they have a catecholamine nucleus (Moini et al. 2021). Neurotransmitters have many functions in regulating mood, arousal, emotion, and cognition, and they are released in both the central nervous system (CNS) and peripheral nervous system (PNS). Their contributions to the field of neuropsychopharmacology, specifically in relation to mood, are highly noteworthy. Serotonin has important functions in regulating mood, emotions, and memory, while dopamine is crucial for mood, motivation, memory, and movements (referred to as the four Ms). Norepinephrine and epinephrine have roles in regulating attention and arousal (Gu et al. 2018).

Astrocytes play a vital role in the survival of neurons in the central nervous system by exerting a neuroprotective effect. They achieve this by forming mitochondria-derived inclusions in aging and deteriorating neurons, as well as by containing reduced glutathione (Dringen 2000; Schipper 2004). Glial fibrillary acidic proteins (GFAP) are over-expressed by astrocytes in response to CNS injury so they serve as markers for neural tissue (Sofroniew and Vinters 2010). Calretinin (CR) is a calcium-binding protein that is made by neurons that buffer excess free Ca^2+^ ions in the neurons to lessen excitotoxicity (Rycerz et al. 2014).

Utilizing naturally existing compounds as neuroprotective drugs is a promising therapeutic approach that reduces harmful damage to the brain. Camellia sinensis, also known as Green tea or GT, is a valuable source of essential hexogen antioxidants. These antioxidants are not naturally produced by our systems and play a key role in combating the free radicals that are produced internally (Aboulwafa et al. 2019). Green tea’s antioxidants are mostly made up of polyphenols, caffeine, minerals, and minute amounts of other substances (Prasanth et al. 2019; Vishnoi et al. 2018). The main catechin compounds found in green tea are (-) Epigallocatechin Gallate (EGCG), (-) Epigallocatechin (EGC), (-) Epicatechin Gallate (ECG), and (-) Epicatechin (EC). Antioxidants are substances that possess the ability to inhibit the oxidation process of other molecules. Guard cells provide protection against the detrimental effects of reactive oxygen species and have the ability to halt chain reactions that cause cellular damage. Green tea catechins mitigate oxidative stress responses associated with cardiovascular and non-alcoholic fatty liver disorders through their dual role as an antioxidant and an anti-inflammatory agent (Abunofal and Mohan 2022; Velayutham et al. 2008). Green tea use is linked to a lower risk of death from all causes and may help prevent Parkinson’s disease, cognitive decline, and osteoporosis (Huang et al. 2020; Li et al. 2022). In conclusion, free radicals can be neutralized by the antioxidants included in green tea, which may help lessen or perhaps prevent some of the harm they cause.

The primary objective of this study is to investigate the harmful effects on the nervous system and changes in metabolism resulting from the prolonged use of monosodium glutamate in adult male albino rats. Additionally, this study aims to assess the potential protective effects of Camellia sinensis and explore the underlying mechanism by which it may counteract the toxic effects of monosodium glutamate when administered together.

## Materials and methods

### Chemicals

The monosodium glutamate salt and Camellia sinensis (green tea) leaves were acquired from Sigma-Aldrich, USA. Subsequently, MSG and Camellia sinensis were supplied following their dissolution in distilled water. All chemical substances utilized were of a superior analytical quality.

### Animals and ethical considerations

Thirty-two mature male albino rats weighing 150 ± 20 g were obtained from the National Research Centre Animal House (Dokki, Giza, Egypt) and were kept in standard polypropylene cages under standard environmental conditions with equal light-dark cycles. Rats were adapted for 1 week and were fed rat normal pellet diet and water *ad libitum*, before the beginning of the experiment. The experiment was approved by the Ethics Committee of the Faculty of Medicine, Ain Shams University [IRB No. FMASU (R156/2023)] and followed the guidelines of the International Council on Harmonization (ICH) and the Islamic Organization of Medical Sciences (IOMS), the United States Office for Human Research Protections, and the United States Code of Federal Regulations and operates under Federal Wide Assurance No. FWA 000017585.

### Experimental design

Thirty-two adult male rats were randomly divided into four equal groups (*n* = 8); Group 1; normal control receiving normal saline (1 ml/rat/day) Group 2 received Green tea extract (1.5 ml/rat/day) (Ahmed 2016). Group 3 administered MSG (600 mg/kg /day) (Tawfik and Al-Badr 2012). Group 4; a combination of MSG (600 mg/kg/day) and GT (1.5 ml/rat/day). All groups were treated orally for 28 days. At the end of the experiment, rats were deprived of food with free access to water overnight and then blood samples were collected from the retroorbital plexus under anesthesia (ketamine 40 mg/kg) into clean and dry centrifuge tubes for the separation of serum then stored at -80ºC for biochemical assay of glutathione peroxidase (GP_X_), nitric oxide (NO), total antioxidant capacity (TAC), dopamine, 3,4-Dihydroxyphenylacetic acid, serotonin (5-HT), 5-Hydroxyindoleacetic acid (5-HIAA), and norepinephrine (NE). Finally, all rats were sacrificed by decapitation and brains were fixed in 10% formol saline solution for seven days followed by dehydration, clearing, and mounting in paraffin blocks. The brain tissues were dissected into right and left halves for histological and immunohistochemical studies.

### Assessment of oxidative stress

#### Glutathione peroxidase activity (GPx)

The GPx activity was assessed indirectly by relying on the enzymatic recycling of oxidized glutathione (GSSG) back to its reduced state by glutathione reductase, following the reduction of an organic peroxide by c-GPx. The process of converting NADPH to NADP + is accompanied by a reduction in the amount of light absorbed at a wavelength of 340 nm (A340). This change in absorbance can be used as a method to measure the activity of the GPx enzyme using a spectrophotometer. The activity of GPx was determined based on the instructions provided by the manufacturer (Biodiagnostic, Giza, Egypt). The enzyme activities were expressed as Mu/ml.

#### Nitric oxide (NO)

In the acid medium and the presence of nitrite, the formed nitrous acid diazotize sulphanilamide and the product are coupled with N-(1–naphthyl) ethylenediamine. The resulting azo dye has a bright reddish–purple color which can be measured at 540 nm. NO concentration was measured according to the manufacturer’s instruction (Biodiagnostic, Giza, Egypt) and was expressed as µmol/L.

#### Total antioxidant capacity (TAC)

The assessment of the antioxidant capacity is carried out by the reaction of the sample’s antioxidant with a specific amount of exogenous hydrogen peroxide (H_2_O_2_). The antioxidants in the sample eliminate a certain amount of the provided hydrogen peroxide which is detected calorimetrically at 505 nm by an enzymatic reaction that involves the conversion of 3,5, dichloro − 2– hydroxy benzenesulfonate to a colored product. Total antioxidant capacity was estimated according to the manufacturer’s instruction (Biodiagnostic, Giza, Egypt) and was expressed as Mm/L.

### Assessment of neurotransmitters

The levels of dopamine (DA), 3,4-Dihydroxyphenylacetic acid (DOPAC), serotonin (5-HT), 5-Hydroxyindoleacetic acid (5-HIAA), and norepinephrine (NE) were evaluated in the serum utilizing HPLC (Thermo Dionex Ultimate 3000) (Koshiishi et al. 1998; Sakuma et al. 1987). In a brief procedure, 100 µL of the serum sample is placed in a 1.5-mL microcentrifuge tube on crushed ice. 1.5 µL of ice-chilled 7 M perchloric acid (PCA) was added to make a final PCA concentration of 0.1 M. The sample was then capped and vortexed immediately after the addition of PCA to avoid gelling of the protein in the sample then centrifuged at 14,000 rpm (18,000 × g) for 15 min, 4 °C then the supernatant was transferred into a clean 1.5-mL microcentrifuge tube and centrifuged again. The supernatant was then ready for HPLC assay. The sample was separated using a Zobrax XDB C18 column per these instructions. Each monoamine concentration in the sample was identified in comparison to the standard and was calculated as µg per ml serum.

### Histological and immunohistochemical studies

The sections of the right halves were cut at 5 μm and were stained with hematoxylin and eosin (H&E), toluidine blue, and silver stains by (Glees and Marseland’s silver) (G&M) technique to evaluate the structures of the hippocampus area CA1, dentate gyrus, and the cerebellum.

The sections of left halves were mounted on positively charged slides for the Avidin-Biotin Peroxidase technique using primary antibodies for Glial Fibrillary Acidic Protein (GFAP) (1:1000) (cat# AB5804; Sigma-Aldrich, St. Louis, MO, USA) for detection of astrocytes. Sections were incubated with biotinylated anti-rabbit secondary antibody (1:2000) (cat# BA-9200; Vector Lab, Burlingame, CA, USA) for 30 min.

To demonstrate the immunoreactivity of calretinin (CR) in neurons, an indirect immunohistochemical peroxidase–anti peroxidase reaction (PAP) was achieved. Brain sections were treated with 0.4% H2O2 at room temperature for 30 min to prevent the endogenous peroxidase. After rinsing in 0.5 M-Tris aminomethane (TRIS) buffer (TBS, pH = 7.6), the sections were incubated in normal goat serum at room temperature for 20 min to eliminate background staining. A set of antibodies and reagents (Sigma-Aldrich, St. Louis, MO, USA) were diluted with 0.5 MTBS according to the producer’s recommendations and were used to conduct an immunohistochemical PAP reaction. The primary antibody was a specific monoclonal rabbit anti-CR antibody (incubation for 48 h at 4°C), whereas the secondary antibody was a monoclonal goat anti-immunoglobulin G (IgG) antibody (Sigma-Aldrich). Finally, the monoclonal peroxidase–anti peroxidase complex was applied. Calretinin is a calcium-binding protein made in neurons and has a neuroprotective effect by buffering excess free Ca2 + ions in the neurons to reduce excitotoxicity. Immunostaining was performed by Avidin Biotin Peroxidase technique. All sections were stained by incubation with 3,3’-diaminobenzidine (DAB), solution (purchased from DAKO, Denmark) for 10 min. Finally, the slides were counterstained with hematoxylin, dehydrated, cleared, and mounted. Negative controls were processed according to the same protocol, except for the use of the primary antibody (Suvarna et al. 2013).

#### Morphometric study

Samples were analyzed by using a Leica DM2500 microscope with a built-in camera (Wetzlar, Germany). All images were digitally acquired using an image analyzer Leica Q Win V.3 program (Wetzlar, Germany) installed on a computer in the Histology Department at the Faculty of Medicine, Ain Shams University. Specimens from all groups were subjected to morphometric study. Measurements were taken from six stained different sections obtained from each group and six non-overlapped high-power fields were examined in each section to measure the mean number of viable neurons, the optical density of toluidine blue stain (to detect Nissl granules density), the mean number of cells with GFAP immune reaction, and the mean number of calretinin-positive cells. All parameters were measured in neurons in the CA1, dentate gyrus, and cerebellar cortex.

### Statistical study

Statistical analysis was carried out by one-way analysis of variance (ANOVA) followed by the Tukey-Kramer test for multiple comparisons. *P < *0.05 is considered significant. GraphPad Prism software version 9 (ISI® software, USA) was utilized for the analysis of data and graph presentations.

## Results

### Assessment of oxidative stress

#### Glutathione peroxidase (GPx) & nitric oxide (NO)

**Fig. 1:**
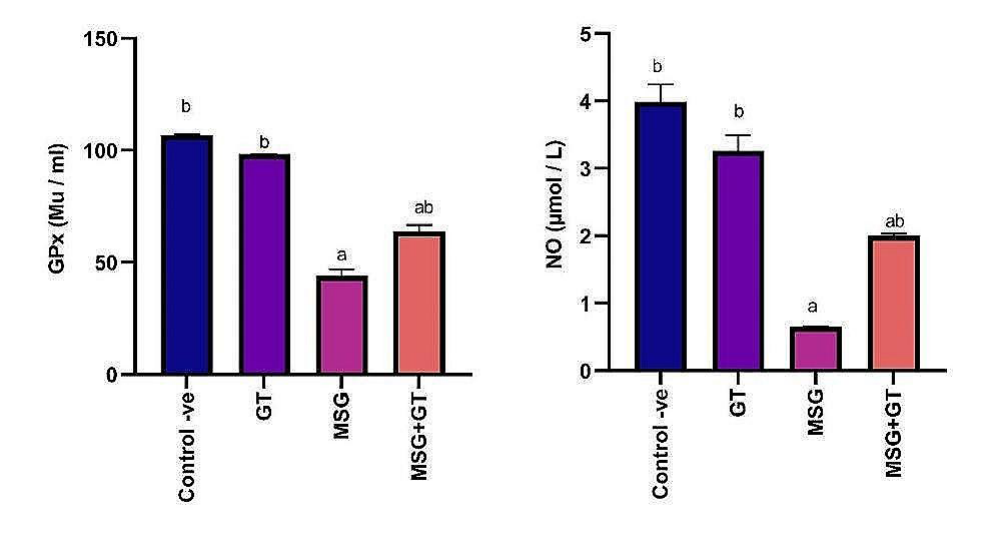
Data are represented as mean ± SE (*n* = 8). ^a^*P* < 0.05 is significant as compared to control group & ^b^*P* < 0.05 is significant as compared to MSG treated group by one-way ANOVA followed by Tukey’s post hoc test. MSG: monosodium glutamate, GT: green tea SE: Standard error, ANOVA: Analysis of variance, GPx: glutathione peroxidase, and NO: nitric oxide

Glutathione peroxidase is a selenium-containing enzyme that is essential for shielding cells from oxidative damage brought on by free radicals and reactive oxygen species. Nitric oxide (NO) is a potent antioxidant that breaks chains in the context of free radical-mediated lipid oxidation (LPO). It functions as a sacrificial chain-terminating antioxidant by reacting quickly with peroxyl radicals. Oral administration of GT for 28 days for rats did not show any significant (*P *˂ 0.05) change in GPx (98.26 ± 0.11 Mu/ml) and NO (3.26 ± 0.23 µmol/L) as compared to the control -ve group (107 ± 0.15 Mu/ml) and (3.99 ± 0.26 µmol/L) respectively. On the other hand, oral administration of MSG daily for 28 days significantly (*P *˂ 0.05) reduced GPx (44.04 ± 2.89 Mu/ml) and NO (0.65 ± 0.01 µmol/L) showing a (2.43&6) fold inhibition respectively as compared to control-ve group. Our results also revealed that concurrent treatment of GT with MSG for 28 days had significantly (*P *˂ 0.05) increased GP_X_ (63.79 ± 2.86 Mu/ml) and NO (2 ± 0.04 µmol/L) that were inhibited via MSG administration exhibiting a (1.45&3.07) fold increase respectively as compared to MSG group. Data are depicted in Fig. 1.

#### Total antioxidant capacity (TAC)

**Fig. 2:**
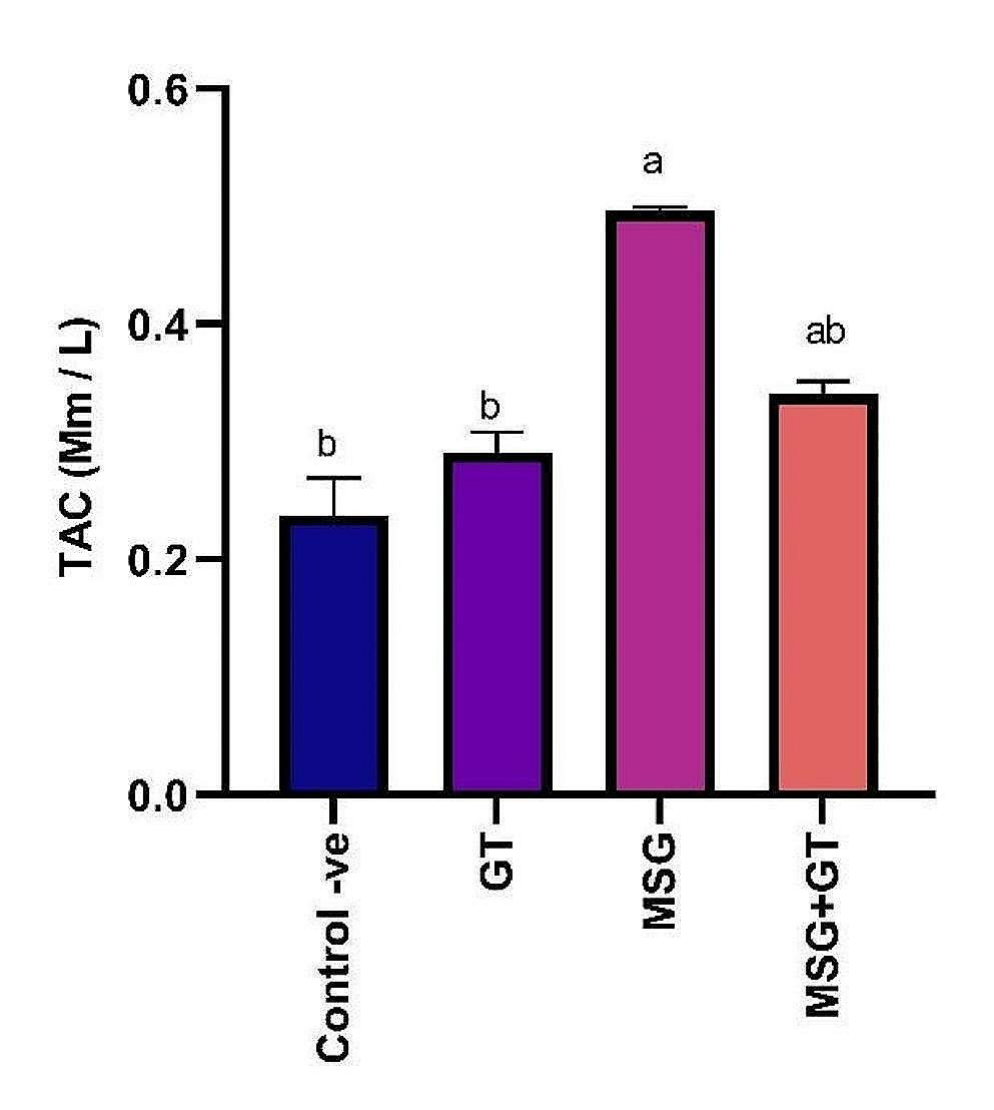
Data are represented as mean ± SE (*n* = 8). ^a^*P* <  0.05 is significant as compared to control group & ^b^*P* < 0.05 is significant as compared to MSG treated group by one-way ANOVA followed by Tukey’s post hoc test. MSG: monosodium glutamate, GT: green tea, SE: Standard error, ANOVA: Analysis of variance, and TAC: total antioxidant capacity

Total antioxidant capacity (TAC) is a measure of the cumulative action of all the antioxidants present in plasma and body fluids. Oral administration of GT for 28 days for rats did not show any significant (*P *˂ 0.05) change in TAC (0.29 ± 0.017 Mm/L) as compared to control -ve group (0.24 ± 0.03 Mm/L). On the other hand, oral administration of MSG daily for 28 days significantly (*P *˂ 0.05) elevated TAC (0.49 ± 0.04 Mm/L) showing a 2.04-fold increase as compared to control-ve group. Our results also revealed that concurrent treatment of GT with MSG for 28 days had significantly (*P* ˂ 0.05) reduced TAC (0.34 ± 0.01 Mm/L) that was inhibited via MSG administration exhibiting a 1.44-fold inhibition as compared to MSG group. Data are depicted in Fig. 2.

### Assessment of neurotransmitters

#### Dopamine (DA) and 3,4-Dihydroxyphenylacetic acid (DOPAC)

**Fig. 3:**
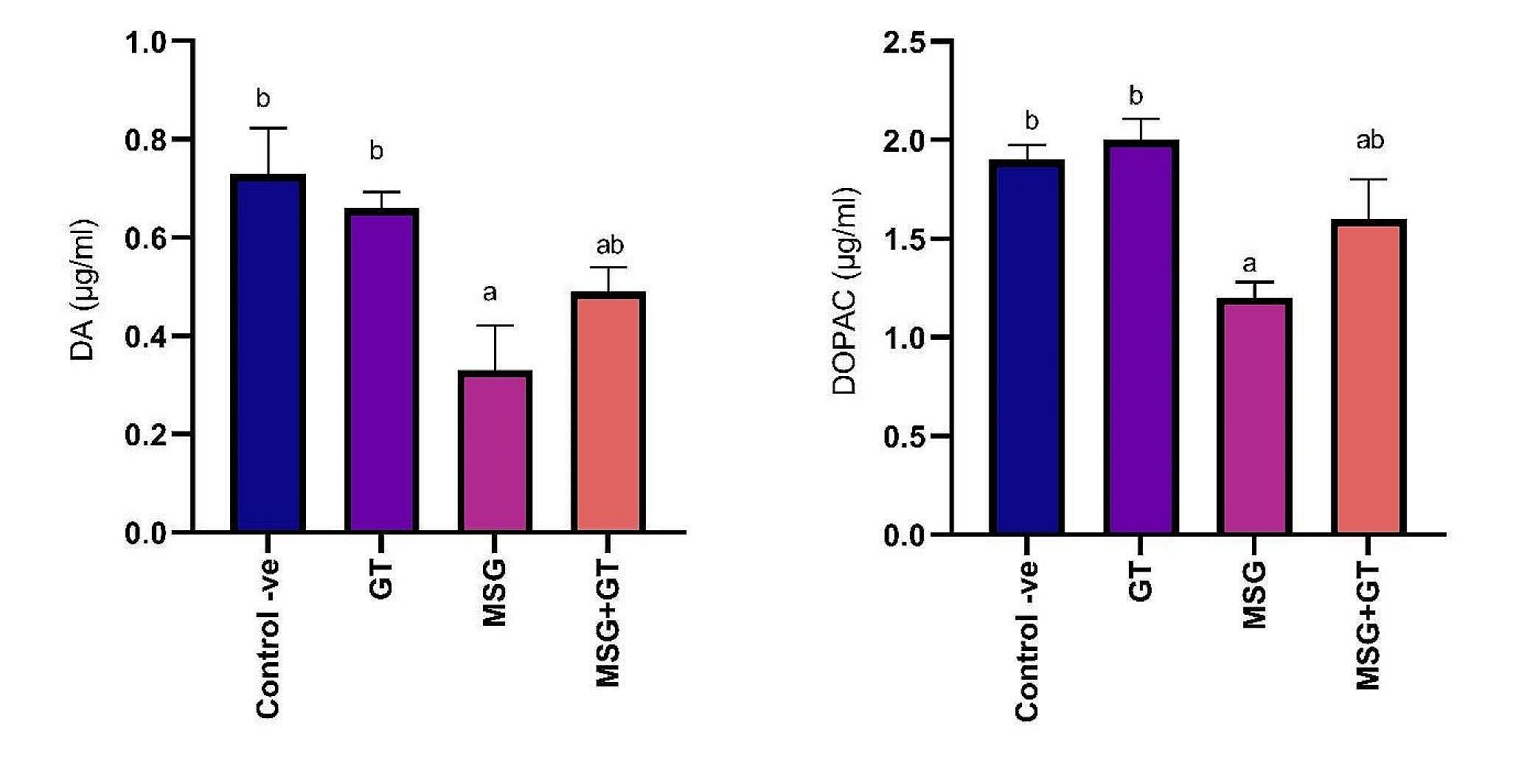
Data are represented as mean ± SE (*n* = 8). ^a^*P* < 0.05 is significant as compared to control group & ^b^*P* <  0.05 is significant as compared to MSG treated group by one-way ANOVA followed by Tukey’s post hoc test. MSG: monosodium glutamate, GT: green tea, SE: Standard error, ANOVA: Analysis of variance, DA: dopamine, and DOPA: 3,4-Dihydroxyphenylacetic acid

Dopamine (DA) is a neurotransmitter that influences reward-motivated behavior, motor control, and mood regulation. It can be metabolized into one of three substances, including 3,4-dihydroxyphenylacetic acid (DOPAC). DOPAC is a phenolic acid and a neuronal metabolite of dopamine which is generated when dopamine is oxidized via monoamine oxidase (MAO). Oral administration of GT for 28 days for rats did not show any significant (*P* ˂ 0.05) change in DA (0.66 ± 0.03 µg/ml) and DOPAC (20 ± 1.1 µg/ml) as compared to control -ve group (0.73 ± 0.09 & 1.9 ± 0.07 µg/ml) respectively. On the other hand, oral administration of MSG daily for 28 days significantly (*P* ˂ 0.05) reduced DA and DOPAC (0.33 ± 0.09 & 1.2 ± 0.08 µg/ml) respectively showing a (2 & 1.67) fold inhibition respectively as compared to control-ve group. Our results also revealed that concurrent treatment of GT with MSG for 28 days had significantly (*P* ˂ 0.05) increased DA (0.49 ± 0.05 µg/ml) and DOPAC (1.6 ± 0.22 µg/ml) that were declined via MSG administration exhibiting a (1.48 & 1.33) fold increase respectively as compared to MSG group. Data are depicted in Fig. 3.

#### Serotonin (5-HT) and 5-Hydroxyindoleacetic acid (5-HIAA)

**Fig. 4:**
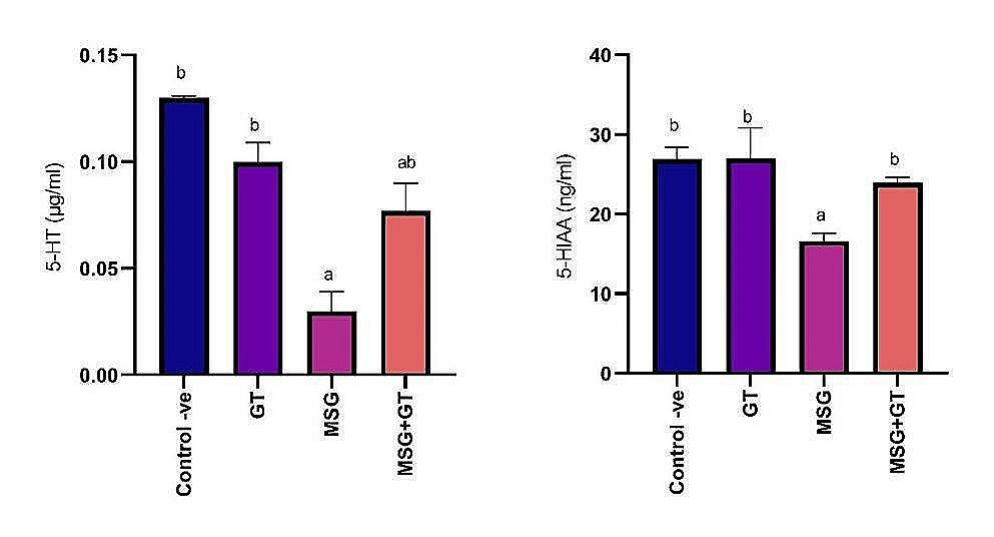
Data are represented as mean ± SE (*n* = 8). ^a^*P* < 0.05 is significant as compared to control group & ^b^*P* < 0.05 is significant as compared to MSG treated group by one-way ANOVA followed by Tukey’s post hoc test. MSG: monosodium glutamate, GT: green tea, SE: Standard error, ANOVA: Analysis of variance, 5-HT: serotonin, and 5-HIAA: 5-Hydroxyindoleacetic acid

Serotonin (5-HT) is a hormone that plays a crucial role in nerve impulses’ transmission and affects mood and sleep. It is metabolized within the liver into 5-hydroxyindoleacetic acid (5-HIAA). No significant (*P* ˂ 0.05) changes in comparison with control -ve group were recorded after oral administration of GT for 28 days to albino rats in 5-HT and 5-HIAA (0.1 ± 0.01 µg/ml & 27 ± 3.8 ng/ml) respectively as compared to control -ve group (0.1 ± 0.001 µg/ml & 26.9 ± 1.5 ng/ml) respectively. While oral administration of MSG daily for 28 days significantly (*P* ˂ 0.05) reduced 5-HT and 5-HIAA (0.03 ± 0.009 µg/g & 16.6 ± 1.0 ng/ml) respectively showing a (1.67 & 1.62) fold inhibition respectively as compared to control -ve group. Additionally, results showed that the combinational administration of GT with MSG for 28 days had significantly increased 5-HT (0.077 ± 0.013 µg/ml) and 5-HIAA (23.9 ± 0.72 ng/ml) that were declined via MSG administration exhibiting a (2.56 & 1.39) fold increase respectively as compared to MSG group. Data are represented in Fig. 4.

#### Norepinephrine (NE)

**Fig. 5:**
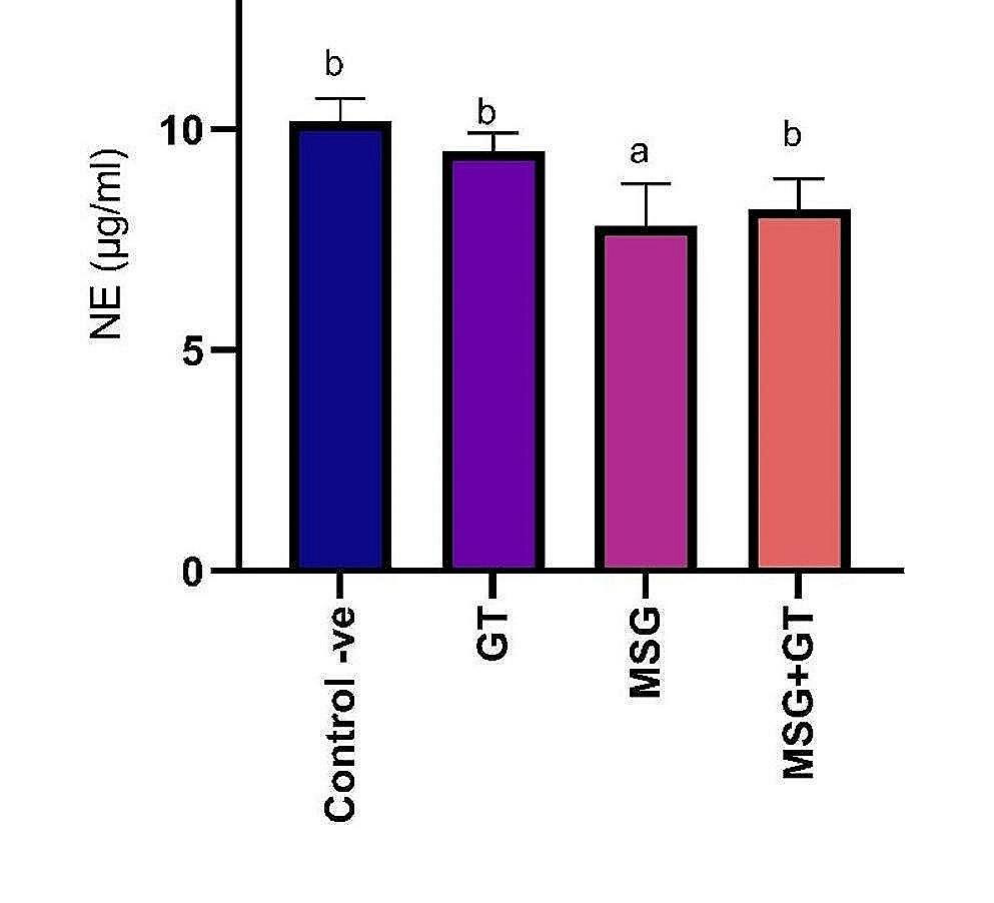
Data are represented as mean ± SE (*n* = 8). ^a^*P* < 0.05 is significant as compared to control group & ^b^*P* < 0.05 is significant as compared to MSG treated group by one-way ANOVA followed by Tukey’s post hoc test. MSG: monosodium glutamate, GT: green tea, SE: Standard error, ANOVA: Analysis of variance, and NE: norepinephrine

The chemical norepinephrine also referred to as noradrenaline, functions as a neurotransmitter and a hormone. Norepinephrine functions as a neurotransmitter, assisting in the transfer of nerve signals from one nerve cell, muscle cell, or gland cell to another across nerve ends. When under stress, it helps to maintain blood pressure by increasing alertness, arousal, attention, and cognitive performance as well as by constricting blood vessels. Oral administration of GT for 28 days for rats did not show any significant (*P* ˂ 0.05) change in NE (9.5 ± 0.42 µg/ml) as compared to control -ve group (10.2 ± 0.52 µg/ml). On the other hand, oral administration of MSG daily for 28 days significantly (*P* ˂ 0.05) reduced NE (7.8 ± 0.97 µg/ml) showing a 1.31-fold inhibition as compared to control-ve group. Our results also revealed that concurrent treatment of GT with MSG for 28 days had significantly (*P* ˂ 0.05) increased NE (8.2 ± 0.68 µg/ml) that was inhibited via MSG administration exhibiting a 1.05-fold increase as compared to MSG group. Data are depicted in Fig. 5.

### Histological and immunohistochemical studies

**Fig. 6:**
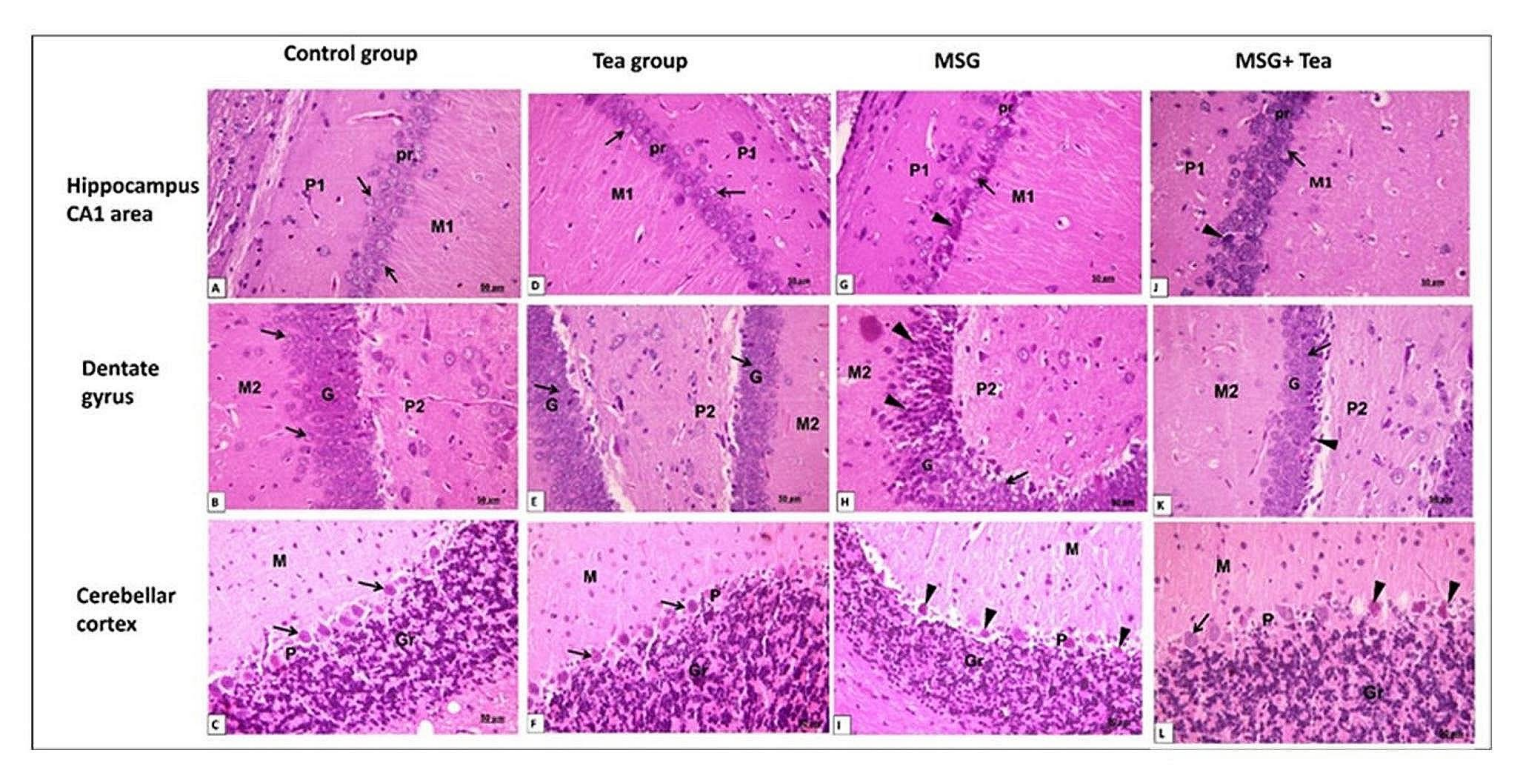
**A**–**L** Photomicrographs showing H&E-stained sections (x400, scale bar: 50 μm) of hippocampal CA1 area, dentate gyrus (DG) and cerebellar cortex (CC). **A**–**F** The control and tea groups both appeared with normal cell structure and arrangement. All nerve cells have large vesicular nuclei (↑). In CA1, the cells are arranged in the pyramidal cell layer (Pr) with some neuroglial cells in molecular (M1) and polymorphic (P1) cell layers. In DG, the granule cells are arranged in the granular cell layer (G) with some neuroglial cells in molecular (M2) and polymorphic (P2) cell layers. CC shows the three layers; outer molecular (M), middle Purkinje (P), and inner granular (Gr) layers. Purkinje cells (↑) are seen in one row with large flask-shaped cell bodies, central vesicular nuclei, and basophilic cytoplasm. The granular layer contains small closely packed granule cells. The molecular layer contains scattered small cells. **G**–**I** The MSG group shows shrunken degenerated neurons (arrowheads) with pyknotic nuclei and increased perineural spaces. Some pyramidal cell bodies of CA1 appeared flame-like with pointed ends (arrowheads). **J**–**L** MSG + GT treated group displayed regularly arranged neurons with vesicular nuclei (↑). Few cells are still shrunken showing deeply stained pyknotic nuclei (arrowheads)

*Examination of the H&E* sections of the hippocampus cortex (CA1 and dentate gyrus (DG), and cerebellar cortex (CC) showed the control & tea groups with normal cell structure and arrangement; the hippocampus area CA1 and dentate gyrus are formed of three layers. CA1 is formed of molecular, pyramidal, and polymorphic cell layers. DG is formed of molecular, granular, and polymorphic cell layers. The main cell layer of the hippocampus proper is the pyramidal cell layer (PL) which consists of closely packed pyramidal cells with large central vesicular nuclei and basophilic cytoplasm. The main cell layer of the DG is the granular layer (Gr) which is formed of closely packed rounded small granule cells containing large vesicular nuclei. The polymorphic layer (PL) displayed deeply, and lightly stained nuclei of the glial cells. The molecular layer (ML) showed a loose appearance. Both the polymorphic layer and the molecular layer present CA1 and DG display the same structures. The cerebellar cortex has three layers; outer molecular, middle Purkinje, and inner granular layers. Purkinje cells were seen in one row with large flask-shaped cell bodies, central vesicular nuclei, and basophilic cytoplasm. The granular layer contained small closely packed granule cells. The molecular layer contained scattered small cells Fig. 6A–F. While the MSG group showed shrunken degenerated neurons with pyknotic nuclei and increased perineural spaces Fig. 6G–I. In groups treated with MSG and GT, neurons appeared regularly arranged with vesicular nuclei. Few cells were still shrunken showing deeply stained pyknotic nuclei Fig. 6J–L.

These findings were confirmed with statistical analysis where there is a significant increase in the mean number of degenerated cells in all areas in the MSG group in comparison to the other groups, while there is a non-significant increase in the group of MSG + GT compared to the control group Table 1.

**Table 1:** The effect of monosodium glutamate (MSG) and green tea (GT) on the histological and immunohistochemical changes in the hippocampus cortex (CA1 dentate gyrus (DG), and the cerebellar cortex (CC)

Groups	Control	GT	MSG	MSG + GT
CA1				
• Number of degenerated cells	2.4 ± 0.24	2.2 ± 0.73	22 ± 1.26^ac^	4 ± 0.7^b^
• Optical density of Nissl’s granules	91.47 ± 0.14	91.88 ± 0.24	76.93 ± 0.54^ac^	89.73 ± 2.4^b^
• Number of GFAP-positive cells	8.8 ± 0.58	7 ± 0.31	23 ± 1.8^ac^	10 ± 0.7^ac^
• Number of calretinin-positive cells	2.2 ± 0.37	1.2 ± 0.31	9.3 ± 0.58^ac^	4 ± 0.7^bc^
Dentate gyrus				
• Number of degenerated cells	5 ± 0.7	4.2 ± 0.86	26.4 ± 1.02^ac^	8 ± 0.5^b^
• Optical density of Nissl’s granules	91.53 ± 0.21	92.25 ± 0.17	76.17 ± 0.53 ^ac^	85.59 ± 0.12^abc^
• Number of GFAP-positive cells	7.6 ± 0.74	7.2 ± 0.37	24.6 ± 0.92^ac^	9 ± 0.54 ^b^
• Number of calretinin-positive cells	2 ± 0.21	1.4 ± 0.5	10.6 ± 0.4 ^ac^	4 ± 1.5^bc^
Cerebellar cortex				
• Number of degenerated cells	3 ± 0.44	2.8 ± 0.4	10.4 ± 0.5^ac^	3.4 ± 0.5 ^b^
• Optical density of Nissl’s granules	97.54 ± 0.37	98.25 ± 0.49	77.01 ± 0.69^ac^	92.31 ± 0.07^abc^
• Number of GFAP-positive cells	7.4 ± 0.5	6.3 ± 0.52	22.7 ± 1.02 ^ac^	8.4 ± 0.52 ^b^
• Number of calretinin-positive cells	9.2 ± 0.73	8.8 ± 0.37	22.6 ± 0.81 ^ac^	14 ± 0.54^abc^

Data are mean ± SE (*n* = 8)

*MSG* monosodium glutamate, *GT* green tea by one-way ANOVA with Tukey’s post hoc test, *SE *Standard error, *ANOVA* Analysis of variance

^a^*P* < 0.05, versus the control group,

^b^*P* < 0.05, versus MSG treated group and

^c^*P* < 0.05, versus teatreated group

**Fig. 7:**
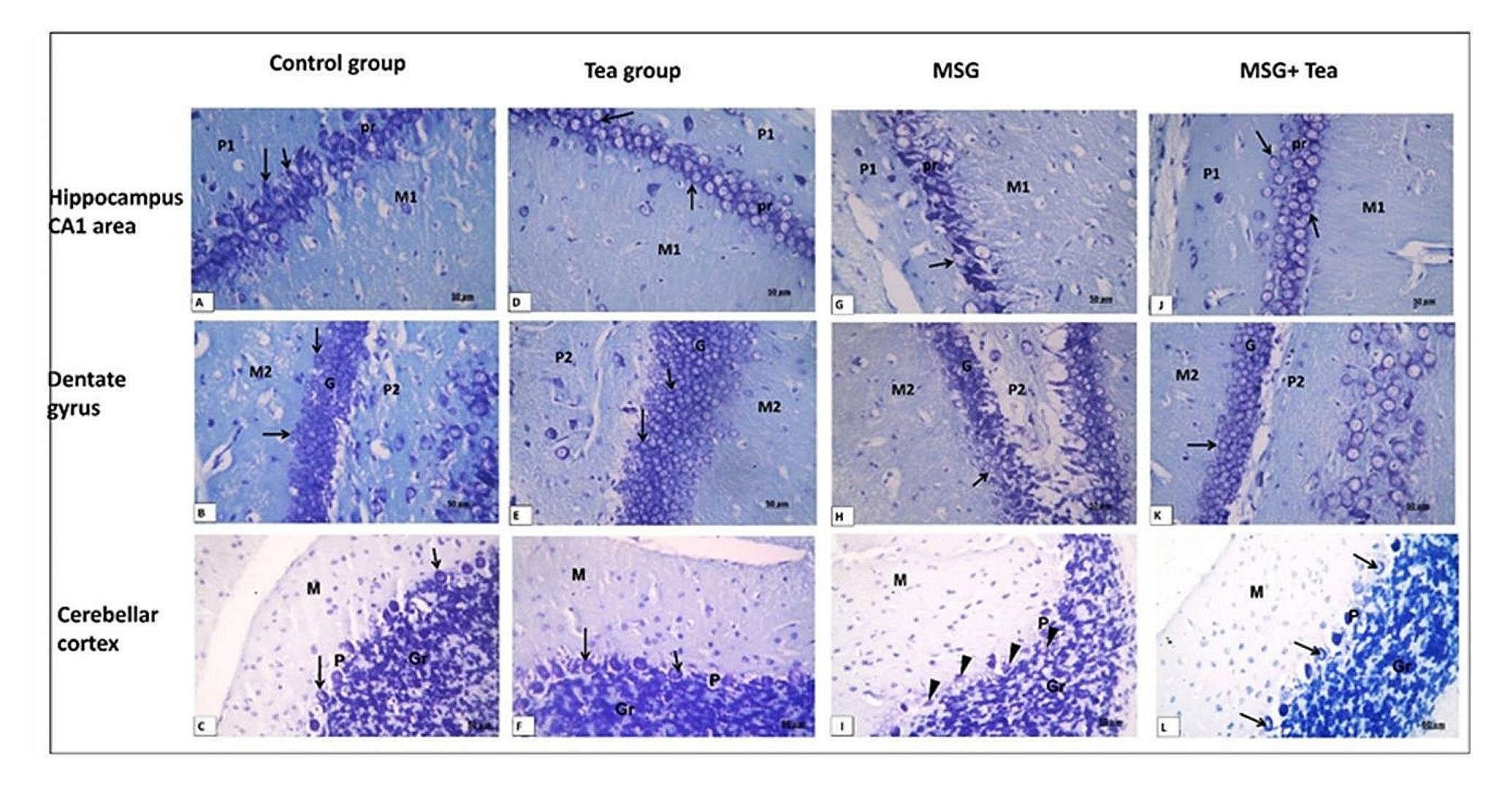
**A**–**L** Photomicrographs showing Toluidine blue stained sections (x400, scale bar: 50 μm) of hippocampal CA1 area, dentate gyrus (DG) and cerebellar cortex (CC). **A**–**F** The control & tea groups show most of the nerve cells with coarse Nissl’s granules surrounding the vesicular nuclei (↑). **G**–**I** In the MSG group, most neurons appear with decreased content of Nissl’s granules (arrowheads) with indistinct nuclei. In CC, Purkinje cells appear with an irregular shrunken outline with loss of one-row organization (arrowheads). **J**–**L** In MSG + GT treated group, most of the the neurons retained their normal architecture (↑)

*Toluidine blue stained* sections of the hippocampal CA1 area, dentate gyrus (DG) and cerebellar cortex (CC) showed nerve cells with coarse Nissl’s granules surrounding the vesicular nuclei in control & tea groups Fig. 7A–F. Most of the neurons in the MSG group appeared with decreased content of Nissl’s granules (chromatolysis) and indistinct nuclei. In CC, Purkinje cells appeared with irregular shrunken outlines with loss of one-row organization Fig. 7G–I. In MSG + GT treated group, most of the neurons retained their normal architecture Fig. 7J–L. A significant decrease in toluidine blue optical density was detected in the MSG group in the CA1 area, dentate gyrus (DG), and cerebellar cortex (CC) compared to the control, tea and (MSG + GT) groups Table 1.

**Fig. 8:**
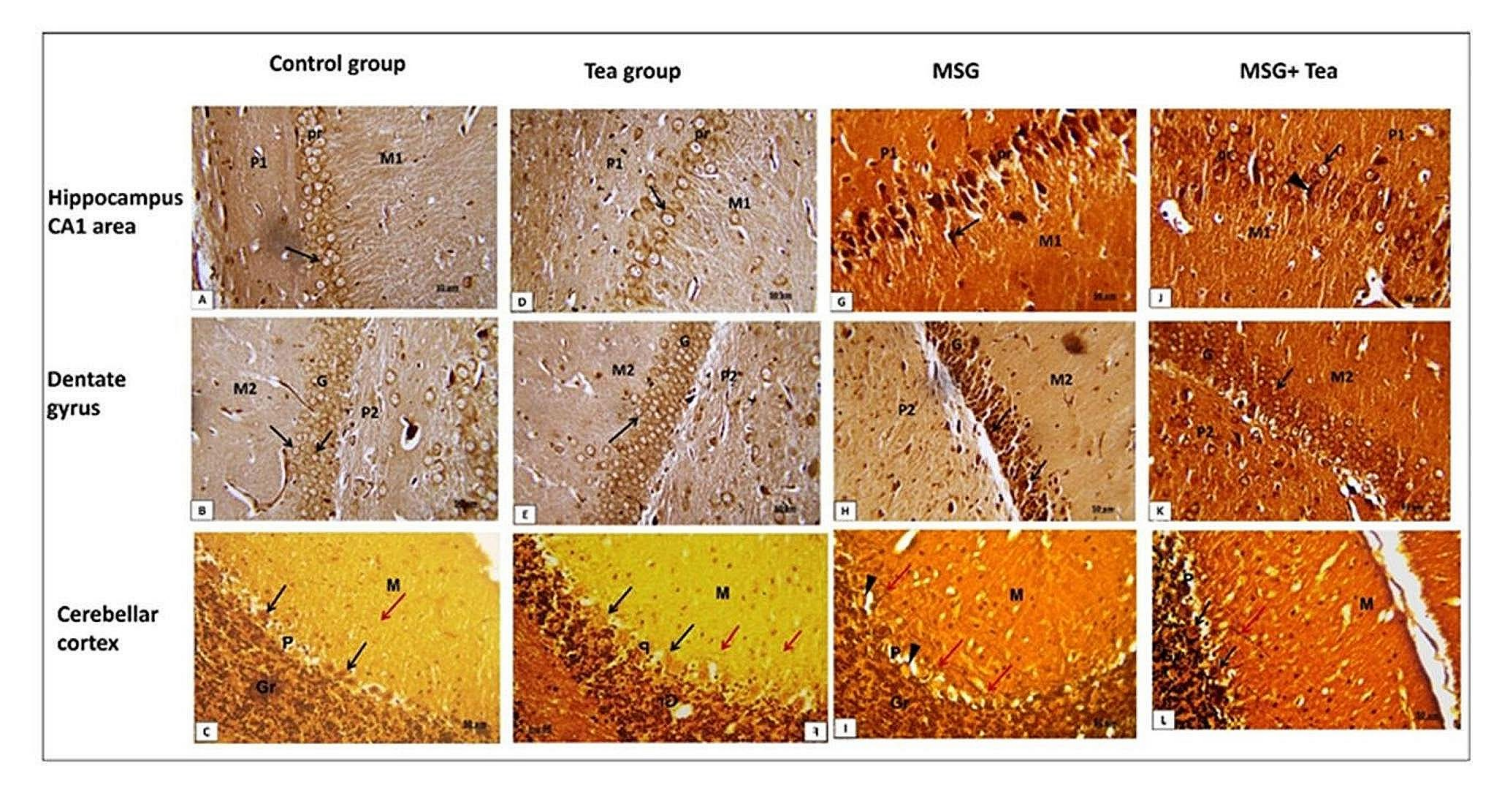
**A**–**L** Photomicrographs showing silver-stained sections (x400, scale bar: 50 μm) of hippocampal CA1 area, dentate gyrus (DG), and cerebellar cortex (CC). **A**–**F** In the control & tea groups, the cytoplasm of all neurons appears with normal distribution of neurofilaments (↑). Nerve fibers appear thin and straight in CC (red arrows). **G**–**I** In the MSG group, most of neurons appear distorted with irregular distribution of neurofilaments which appear as neurofibrillary tangles (arrow heads). In CC, nerve fibers appear thick, wavy, and beaded (red arrows). **J**–**L** In MSG + GT treated group, neurofilaments in most of neurons retained their normal distribution (↑). In CC, the nerve fibers still show some irregularity but less than MSG treated group (red arrows)

*Sections stained with silver* of hippocampal CA1 area, dentate gyrus (DG), and cerebellar cortex (CC) showed that the cytoplasm of all neurons appears with normal distribution of neurofilaments, nerve fibers appeared thin and straight in CC in control and GT groups Fig. 8A–F. In MSG group, most of the neurons were observed to be distorted with an irregular distribution of neurofilaments which appeared as neurofibrillary tangles. In CC, nerve fibers appear thick, wavy, and beaded Fig. 8G–I. MSG + GT treated group showed that neurofilaments in most of the neurons retained their normal distribution. In CC, nerve fibres still showed some irregularity but less than MSG treated group Fig. 8J–L.

**Fig. 9:**
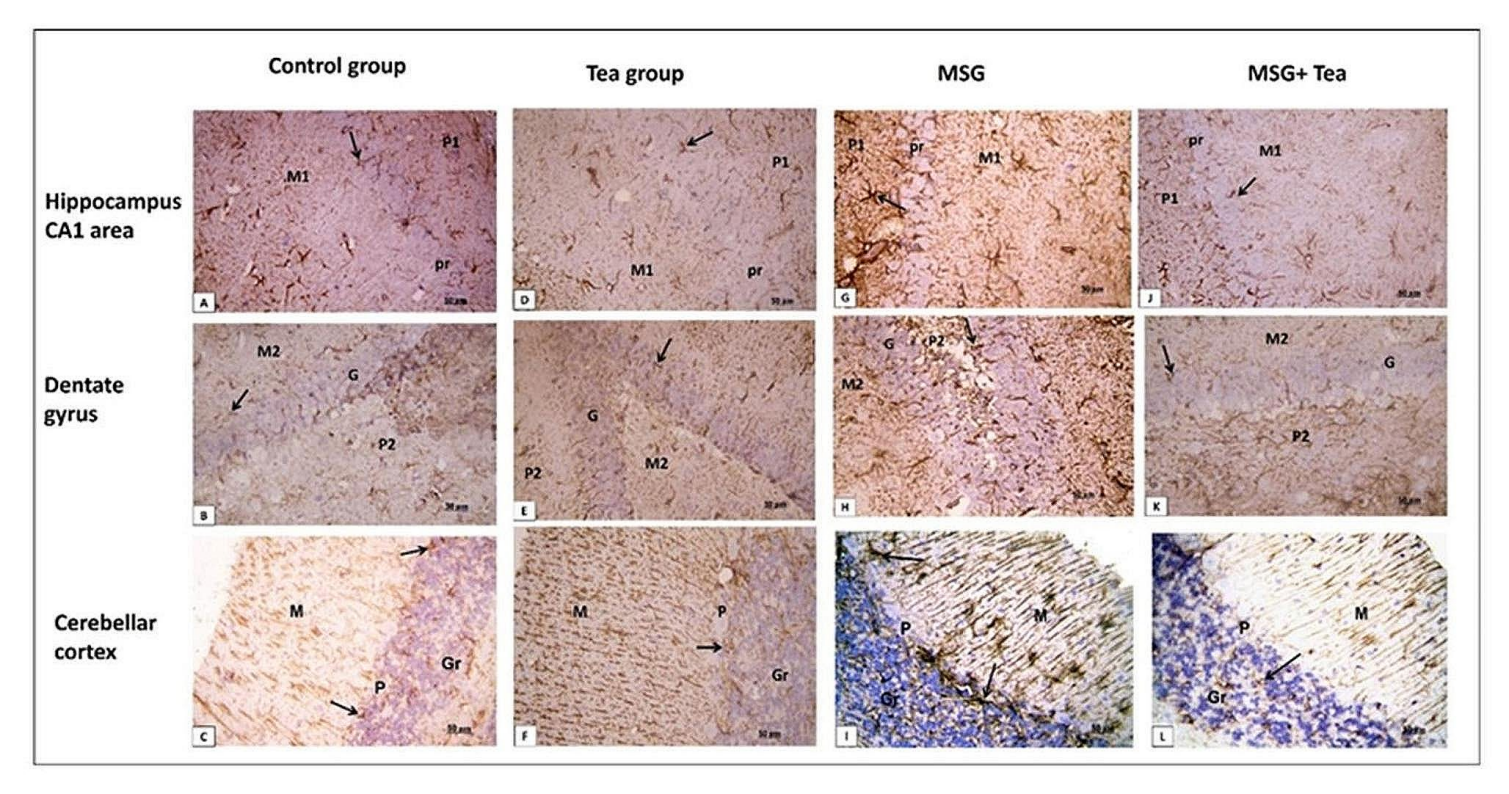
**A**–**L** Photomicrographs showing GFAP immuno-stained sections (x400, scale bar: 50 μm) of hippocampal CA1 area, dentate gyrus (DG), and cerebellar cortex (CC). **A**–**F** The control & tea groups show weak positive brownish immune reactions for GFAP in glial cells (↑). **G**–**I** The MSG group shows a strong positive brownish immune reaction for GFAP in glial cells (↑). **J**–**L** In MSG + GT treated group, a weak GFAP-positive immune reaction can be observed (↑)

*GFAP immuno-stained* sections for hippocampal CA1 area, dentate gyrus (DG), and cerebellar cortex (CC) showed a weak positive brownish immune reaction in astrocytes in the control & tea groups Fig. 9A–F. The MSG group showed a strong positive brownish immune reaction for GFAP and increased numbers of astrocytes Fig. 9G–I. Weak GFAP positive immune reaction and decreased number of astrocytes can be observed in MSG + GT treated group Fig. 9J–L. A significant increase is observed in the number of GFAP-positive cells in the MSG group in all areas compared to the control and tea groups Table 1.

**Fig. 10:**
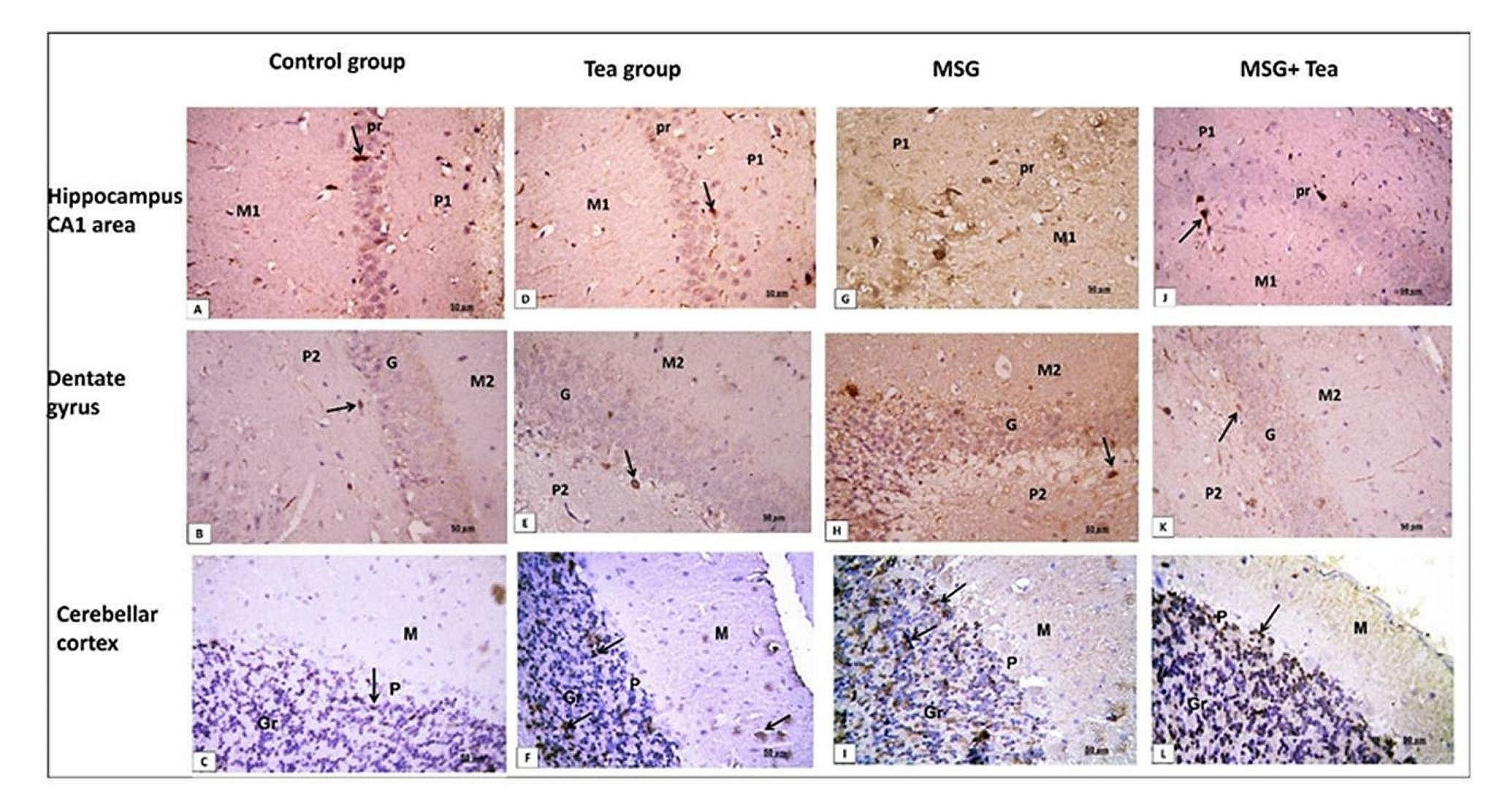
**A**–**L** Photomicrographs showing Calretinin immuno-stained sections (x400, scale bar: 50 μm) of hippocampal CA1 area, dentate gyrus (DG), and cerebellar cortex (CC). **A**–**F** The control & tea groups show few cells with positive brownish immune reactions for calretinin (↑). **G**–**I** The MSG group shows an increased number of calretinin-positive cells (↑). In CC positive reaction is mainly observed in the granular layer (Gr). **J**–**L** In MSG + GT treated group, calretinin-positive cells can be observed but less in number as compared to the control group

*Calretinin immuno-stained* sections of hippocampal CA1 area, dentate gyrus (DG), and cerebellar cortex (CC) showed few numbers of immune-positive brownish cells in control & tea groups Fig. 10A–F. The MSG group showed an increased number of calretinin-positive brownish cells. In CC, a positive reaction is mainly observed in the granular layer Fig. 10G–I. In MSG + GT treated group, calretinin-positive cells can be observed but less in number as compared to MSG group Fig. 10J–L. These data were confirmed by morphometric and statistical analysis Table 1.

## Discussion

MSG is one of the most popular food additives used globally. It accumulates in the brain and causes sustained neurotoxicity, which causes synapse loss and neurodegeneration (Garattini 2000). Glutamate salt is a natural excitatory neurotransmitter, which plays crucial roles in physiological and pathological processes in the central nervous system (Mattson 2008). Neurodegenerative diseases often involve oxidative damage, which occurs due to the chemical reactions between reactive oxygen species (ROS) and specific biomolecules such as proteins, lipids, and nucleic acids (Hussein et al. 2017). The brain’s relatively high concentration of polyunsaturated fatty acids renders it especially vulnerable to membrane lipid peroxidation (Uchida 2003). Excessive glutamate stimulates glutamate receptors and hyper-activates calcium ions to penetrate neurons and flow inside the cell, activating numerous hydrolytic enzymes (Mattson 2008). These enzymes damage DNA, mitochondria, cytoskeleton, membranes, and other cellular components. They also increase oxidative stress and generate free radicals, which ultimately cause cell apoptosis (Fujikawa 2005). The findings of our study showed that the administration of MSG for 28 days resulted in oxidative stress. This led to a notable increase in the Total antioxidant capacity (TAC), which represents the combined effectiveness of all antioxidants in the sample. TAC is considered a crucial measure for evaluating the strength of oxidative/reductive processes (Ghiselli et al. 2000) and remarkable reduction of glutathione peroxidase activity (GPx) which is an enzyme that plays a crucial role in protecting cells from oxidative damage by reducing hydrogen peroxide and lipid peroxides (Muthukumar et al. 2011). Nitric oxide (NO) effectively counteracts the harmful effects of free radicals by inhibiting antioxidant-induced lipid peroxidation (Miles et al. 1996), enhances the potency of reduced glutathione (Matsubara et al. 2005), and up-regulates the enzymatic antioxidants gene expression (Klatt and Lamas 2000). Therefore, a reduction in NO levels in mice treated with MSG suggests a weakening of the cellular defense against nitrosative and oxidative stress. Our data agreed with those recorded by (Saleh et al. 2021) who suggested that that the decrease in GPx levels due to prolonged MSG administration could be caused by excessive consumption or inactivation of GPx by the generation of an abundance of free radicals. These free radicals eventually become hydroxyl radicals, which initiate and propagate lipid peroxidation (Farombi and Onyema 2006). Concomitantly with the inhibition of serum GPx activity, our results also showed that MSG administration caused a significant decrease in serum NO. Our results agreed with a previous study conducted on monosodium-induced testicular toxicity which showed a significant decrease in serum NO (Abd-Elkareem et al. 2021). On the other hand, we recorded a significant increase in TAC levels in serum as a result of MSG administration which agreed with a previous study (Abd-Elkareem et al. 2021) that ascribed this outcome to an adaptive compensating reaction to oxidative instability following the consumption of MSG. Moreover, in order to mitigate the effects of oxidative damage, it was imperative to enhance the overall antioxidant capacity. This was accomplished via stimulating redox-sensitive transcription factors and the subsequent signaling pathways that enhance the body’s natural antioxidant defenses in response to a shift in the redox balance towards the pro-oxidant side (Done and Traustadóttir 2016). The simultaneous treatment of GT with MSG greatly improved the oxidative changes observed in the MSG treated group. The observed outcomes were ascribed to the strong antioxidant properties of GT, which can be attributed to its composition of Epigallocatechin Gallate (EGCG), (-) Epigallocatechin (EGC), (-) Epicatechin Gallate (ECG), and (-) Epicatechin (EC). These compounds have been previously demonstrated to effectively neutralize free radicals and exhibit potential neuroprotective effects (Singh et al. 2016).

The present investigation demonstrated that the oral administration of MSG over a period of 28 days resulted in a significant reduction in the levels of monoamine neurotransmitters, including dopamine (DA), 3,4-dihydroxyphenylacetic acid (DOPAC), serotonin (5-HT), 5-hydroxyindoleacetic acid (5-HIAA), and norepinephrine (NE). Prior studies have yielded comparable findings, demonstrating that the administration of MSG resulted in a reduction of some neurotransmitters and their metabolites, including norepinephrine, serotonin, and dopamine, in the hypothalamic area of the rat brain.(Nakagawa et al. 2000). The depletion observed can be linked to the accumulation of glutamic acid due to excessive consumption of MSG. This accumulation, in turn, leads to neuronal injury and subsequently causes a decrease in neurotransmitter levels (Iuculano et al. 2018; Ubuka 2021). According to earlier research conducted by Odenwald and Turner (2017) (Odenwald and Turner 2017); Pravda (2005) (Pravda 2005), there was notable depletion in the brain cortex of the MSG group’s monoamine levels (NE, DA, and 5-HT). The monoamine neurotransmitter depletion might have also occurred due to oxidative stress induced by MSG (Hussein et al. 2017). The simultaneous ingestion of GT and MSG resulted in a considerable elevation of the decreased levels of neurotransmitters in the bloodstream. Previous studies have shown that GT has the ability to reduce oxidative stress and inflammation, regulate intracellular signaling pathways, aid in metal chelation, and modify the levels of monoamine neurotransmitters and the hypothalamus-pituitary-adrenal (HPA) axis (Chen et al. 2018; Dong et al. 2015; Zhu et al. 2012).

The histology data we obtained support the previous reports of MSG neurotoxicity in rats. This is evidenced by the observed alterations in oxidative stress, nitrosative stress, and neurotransmitter levels in the serum. Our findings indicate that prolonged exposure to MSG resulted in a significant rise in atrophied and damaged neurons with condensed nuclei and expanded spaces surrounding the nerves in the hippocampus (specifically CA1 and dentate gyrus) and cerebellar cortex. Additionally, there was a decrease in the presence of Nissl’s granules (chromatolysis) and unclear nuclei. Purkinje cells in CC exhibited uneven and contracted outlines, accompanied by the loss of their organized arrangement in a single row. The neurodegeneration may be caused by the documented suppression of the antioxidant system and the buildup of glutamic acid in the central nervous system due to excessive consumption of MSG. Prior to this, comparable outcomes had been achieved (Huang et al. 2022). The neurodegeneration was also confirmed by a strong positive brownish immune reaction for GFAP and increased numbers of astrocytes. and increased number of calretinin (CR) -positive brownish cells. These findings agreed with those of Gudiño-Cabrera et al. (2014) (Gudiño-Cabrera et al. 2014) who observed that the treatment of MSG resulted in the enlargement of astrocytes and microglial cells, as well as increased death of neurons in the CA1 and CA3 regions of the hippocampus. However, MSG significantly increased astrogliosis, as evidenced by increased GFAP expression, which indicates increased astrocyte activity after central nervous system (CNS) damage (Demeule et al. 2004). According to a prior study (Rycerz et al. 2014), authors reported that the density of CR-immunopositive cells in the MSG group and their percentage ratio to the density of all cells did not alter significantly following MSG therapy. Still, there was a statistically significant rise in CR-immunopositive cells’ staining intensity. Another study reported upregulation of CR expression in the brain tissue of the MSG-treated group. In that study, there was exitotoxic effects of MSG which resulted from increase in the extracellular brain glutamate concentration followed by over-activation of glutamate receptors. That was associated with Ca^+2^ release from its stores. Increased calcium levels led to mitochondrial over-activation with progressive damage to cell structures including the cytoskeleton, cell membrane, and DNA. Calretinin protein was increased in the brain tissue of the MSG-treated group to play a neuroprotective role by buffering excess calcium and maintained the calcium homeostasis (Hazzaa et al. 2020). The histological and immunohistochemical changes observed in the experiments were mostly reversed when GT was administered alongside MSG. This was attributed to the neuroprotective properties of GT and its ability to restore normal redox activity. It also enhances the restoration of normal redox activity, which helps maintain the cell’s hemostatic response.

## Conclusion

The present study indicates that the oral administration of MSG hampers neural activities in rats that are exposed to it, mostly because it triggers oxidative stress and disrupts neurotransmitters. The neuroprotective efficacy of Camellia sinensis, commonly known as green tea (GT), against MSG-induced neurotoxicity was effectively proven. Our data indicated that GT enhanced the antioxidant system by improving GPx activity, NO levels, and TAC serum levels. Furthermore, it enhanced the modified release of neurotransmitters such as dopamine (DA), dihydroxyphenylacetic acid (DOPAC), serotonin (5-HT), 5-hydroxyindoleacetic acid (5-HIAA), and norepinephrine (NE). After the simultaneous administration of GT with MSG, the degenerative alterations observed in histological and immunohistochemical tests, as shown by the increase in GFAP and CR-immune positive cells, were nearly reversed. Therefore, we have determined that the regular use of GT may hold potential in the treatment of nerve tissue damage caused by MSG.

## Data Availability

No datasets were generated or analysed during the current study.
